# Profiling beneficial phytochemicals in a potato somatic hybrid for tuber peels processing: phenolic acids and anthocyanins composition

**DOI:** 10.1002/fsn3.2100

**Published:** 2021-01-11

**Authors:** Khawla Ben Jeddou, Mariem Kammoun, Jarkko Hellström, Liz Gutiérrez‐Quequezana, Veli‐Matti Rokka, Radhia Gargouri‐Bouzid, Semia Ellouze‐Chaabouni, Oumèma Nouri‐Ellouz

**Affiliations:** ^1^ Laboratory of Plant Improvement and Valorization of Agricultural Resources National Engineering School of Sfax (ENIS) University of Sfax Sfax Tunisia; ^2^ Production Systems Natural Resources Institute Finland (Luke) Jokioinen Finland; ^3^ Food Chemistry and Food Development Department of Biochemistry University of Turku Turku Finland; ^4^ Department of Biology and Geology Preparatory Institute for Engineering Studies of Sfax Sfax Tunisia

**Keywords:** anthocyanins, peels, phenolic acids, potato, somatic hybrid

## Abstract

The purpose of this study was to characterize the peels of a CN1 somatic hybrid obtained from two dihaploid potato lines (Cardinal H14 and Nicola H1) in terms of the health‐promoting phenolic compounds (phenolic acids and anthocyanins). The CN1 hybrid is defined by a pink tuber skin color making it different from the light‐yellow‐skinned “Spunta,” which is the most commonly grown potato cultivar in Tunisia. Oven‐dried peel samples derived from CN1 hybrid and cv. Spunta were ground, and phenolic compounds were extracted with water or methanol for quantification. Lyophilized peels were used for the phenolic acid and anthocyanin analyses. Higher total quantities of phenolic compounds were recovered in methanol extracts compared with water extracts. A slightly higher concentration of phenolic acids (100 mg/100 g DW) was obtained in the lyophilized peels extract of CN1 hybrid than in the cv. Spunta corresponding sample (83 mg/100 g DW). The profiles of the chlorogenic acid isomers were almost identical in both of CN1 hybrid and cv. Spunta. Caffeic acid (CA) and three caffeoylquinic acids (CQAs): 3‐CQA, 4‐CQA, and 5‐CQA, were identified from both genotypes, 5‐CQA being the dominant form in both potatoes. Since the CN1 hybrid has a pink skin color, its anthocyanin profile was also determined. The anthocyanin quantity in the CN1 peels was 5.07 mg/100 g DW, involving six different anthocyanins that were identified within the extract, namely, Pelargonidin‐3‐rutinoside‐5‐glucoside, peonidin‐3‐rutinoside‐5‐glucoside, coumaroyl ester of pelargonidin‐3‐rutinoside‐5‐glucoside, coumaroyl ester of peonidin‐3‐rutinoside‐5‐glucoside, feruloyl ester of pelargonidin‐3‐rutinoside‐5‐glucoside, and feruloyl ester of peonidin‐3‐rutinoside‐5‐glucoside. These results suggest that the peel waste of CN1 somatic hybrid can be considered as a promising source of high‐value compounds for food industry.

## Graphical abstract

The peel waste of the cultivated CN1 somatic hybrid can be considered as a promising source of high‐value compounds for food industry.

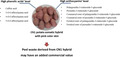

## 1 INTRODUCTION

Potato, the fourth most prominent crop plant after cereals (maize, rice, and wheat), plays an important role in human diet all over the world (Birch et al., 2012; FAOSTAT 2020). Potato is a flexible crop adapted to a wide range of altitudes and various environmental conditions. The overall trend during the latest decades shows a strong increase in potato production especially in Asia, Africa, and Latin America, where the output has risen from less than 30 Mt in the early 1960s to more than 235 Mt in 2016 (FAOSTAT 2020). In 2005, potato production in developing countries exceeded the total output of the developed world. Compared to cereals, potato produces more dry matter, protein and minerals per unit area (Ezekiel et al., 2013). Apart from being a rich source of starch, potatoes also contain various quantities of small molecules and secondary metabolites, which play important roles in a number of bioprocesses (Friedman, 1997). The micronutrients having high bioactivity levels are, however, more enriched in the peels, within the first 1 mm layer from the outside surface of the skin (Friedman, 2006; Friedman et al., 2003; Fritsch et al., 2017; Lewis et al., 1998). Therefore, by‐products from potato processing, such as industrial peel wastes, are a good potential source of high‐value compounds for food industry. Relatively little is still known about the important phytochemicals contained in processing by‐products of potato (Albishi et al., 2013). The beneficial microchemicals in peels are therefore important to be thoroughly characterized before the potential exploitation and commercial value of the manufacturers’ by‐products can be properly evaluated.

One of the particularly important microchemical group in peels is phenolic compounds (polyphenols), which are naturally synthetized by a potato plant for its growth and reproduction, but mainly for its defense response to microbial pathogens, insects, and abiotic stresses (Lattanzio et al., 2006). Phenolic compounds are not only responsible for the major organoleptic characteristics, such as color and taste properties in plant‐derived foods, but they also contribute to health benefits associated with diets rich in vegetables and fruits (Cheynier, 2005). Potato is ranked third most important dietary source of phenolics after apples and oranges (Chen & Chen, 2013; Chun et al., 2005). However, it is occasionally classified even first for the contribution to total polyphenol intake due to the high consumption of potatoes (Brat et al., 2006). Phenolic compounds in potatoes can be present in both soluble‐ (free and soluble esters and soluble glucosides) and insoluble‐bound forms (Albishi et al., 2013; Mattila & Hellström, 2007). Polyphenols consist of multiple phenol units (C_6_H_6_O), and are the basis of the number of phenol rings and structural elements binding rings to one another. They can be classified into four major groups; flavonoids, stilbenes, lignans, and phenolic acids, each having different physical, chemical, and biological properties (Manach et al., 2004). Although the predominant phenolic group in potato is phenolic acids, flavonoids have also been determined in some studies (Furrer et al., 2017; Lewis et al., 1998). Phenolic acids are classified as either hydroxybenzoic acids (e.g., gallic acid, vanillic acid, *p*‐hydroxybenzoic acids) or hydroxycinnamic acids (e.g., caffeic, ferulic, *p*‐coumaric, sinapic acids), and may occur in their acid or as conjugated derivates (as esters) in plants (Clifford, 2000; Manach et al., 2004). Substituted derivatives of hydroxycinnamic acids, called chlorogenic acids (caffeoylquinic acids, CQAs), are the most abundant forms of the phenolic acids in potato (Lewis et al., 1998). The caffeoylquinic acid 5‐CQA (often called chlorogenic acid) is the dominant phenolic compound in white/yellow tubers (Brown, 2005; Malmberg & Theander, 1985), but other isomers, such as 3‐CQA (neochlorogenic acid), 4‐CQA (cryptochlorogenic acid), and free caffeic acids are also found (Andre et al., 2007; Griffiths & Bain, 1997). Low levels of other free phenolic acids, vanillic, protocatechuic, ferulic, sinapic, salicylic, syringic, and *p*‐coumaric acids, have also been reported in potatoes (Lewis et al., 1998; Del Mar Verde Méndez et al., 2004; Andre et al., 2007; Deuβer et al., 2012).

Flavonoids, another group of polyphenols in potato, are divided into six major subclasses; flavones, flavonols, flavanones, catechins (flavanols), anthocyanidins, and isoflavones. Even though most of the flavonoids present in plants are attached to sugars (glucosides), they are occasionally found as aglycones (Ross & Kasum, 2002). In color‐pigmented potatoes, the presence of anthocyanins (glycosylated anthocyanidins) may double the total quantities of all phenolic compounds (Burgos et al., 2013; Lewis et al., 1998). Other flavonoids (flavonols and flavones) are present only in trace amounts, and potatoes are not important dietary sources of such substances. The most abundant flavonols in potato are quercetin‐3‐rutinoside (rutin) and kaempferol‐3‐rutinose (Deuβer et al., 2012). In specific genotypes, such as cv. La Ratte with high rutin concentrations, flavonols can be associated to the accumulation of yellow tuber color (Navarre et al., 2011). In addition, low quantities of flavanones (eriodictoyl and naringenin), catechins, and epicatechins were observed in potato peels (Lewis et al., 1998). Moreover, six anthocyanidins have been reported in plants with vacuolar color pigmentations: pelargonidin (Pg), cyanidin (Cy), delphinidin (Dp), peonidin (Pn), petunidin (Pt), and malvidin (Mv). All of these are detected in various vegetative parts of a potato plant (Harborne, 1960), but in tubers the most common aglycons are pelargonidin, peonidin (in red‐fleshed), petunidin, and malvidin (in purple‐fleshed potatoes) (Lewis et al., 1998). Delphinidin is present only as a minor compound in tubers, and it is rather more concentrated in other parts of a potato plant, such as their flowers (Furrer et al., 2017; Lewis et al., 1998). Cyanidin has recently been identified in both red‐ and purple‐skinned cultivated potato varieties (Furrer et al., 2017; Lachman et al., 2012).

In potato, the color pigments are determined as various types of acylated glucosides, constituting almost 100% of the total anthocyanin content (Fossen & Andersen, 2000). In anthocyanins, conjugates with p‐coumaric acid were detected (Lewis et al., 1998), but anthocyanidins can also be acylated with a moiety of ferulic (Fossen & Andersen, 2000) or caffeic acid (Fossen et al., 2003). In terms of antioxidant content, red and purple‐fleshed potatoes are found to be 2–3 times higher in these substances than white‐ and yellow‐fleshed potatoes (Lachman et al., 2012), and the skin tissues have shown the greatest antioxidant, anti‐inflammatory, and anticancer activities compared to other tuber sections (Brown, 2005; Loo et al., 2016; Reddivari et al., 2007). Observational and intervention studies have also proven the positive effects of polyphenols on the prevention/modulation of metabolic syndrome (Amiot et al., 2016), endothelial dysfunction (Ochiai et al., 2015), hypertension (Medina‐Remón et al., 2015), and cardiovascular and coronary diseases (Yamagata et al., 2015).

In this study, a somatic hybrid derived from protoplast fusion between two dihaploid potato lines was examined for the composition of selected phytochemicals from the peel samples. Potato processing technology worldwide has evolved, and increasing quantities of peel wastes should be considered for their commercial values for nutritional and nonfood uses. The purpose of this study was, therefore, to identify and analyze the composition of beneficial compounds (phenolic acids and anthocyanins) and evaluate the potential value‐added characteristics of a hybrid potato, which expresses a pink tuber skin color. Thereby, the health‐promoting properties of peel wastes can be adequately validated for special market needs.

## 2 MATERIALS AND METHODS

### 2.1 Chemicals

Chlorogenic acid (>98%) and caffeic acid (>98%) were obtained from Sigma Aldrich and cyanidin 3‐O‐glucoside (>96%) was derived from Extrasynthese (Genay, France). Butylated hydroxyanisole (BHA) was from Agros Organics (Geel, Belgium). Phosphoric acid (H_3_PO_4_) was obtained from Merck (Darmstadt, Germany), formic acid (HCOOH) from Sigma Aldrich, and acetic acid (CH_3_COOH) was from Fisher Scientific. Methanol was derived from Fisher Scientific, and acetonitrile from BDH Chemicals.

### 2.2 Sample pretreatment by oven‐drying

Two different potato samples were selected for the study: (a) the potato cultivar Spunta, and (b) an intraspecific somatic hybrid potato line CN1 that originated from a protoplast fusion between two dihaploid potato lines (Cardinal H14 and Nicola H1) (Nouri‐Ellouz et al., 2006). The somatic hybrid line CN1 and cv. Spunta were maintained in vitro by subculturing single‐node cuttings on an MS (Murashige & Skoog, 1962) basal medium supplemented with sucrose (30 g/L) and solidified with agar (8 g/L). Minitubers from in vitro plantlets of the CN1 hybrid and cv. Spunta were grown in the greenhouse at the National Engineering School of Sfax (ENIS, Tunisia), and then, tubers of the second generation were produced in the field using the current cultivation process applied in Tunisia. Field grown tubers of CN1 hybrid and cv. Spunta were washed with tap water and then peeled using a stainless steel knife. The resulting potato peels were dried in a heat oven at 50°C for 48 hr, ground using a Moulinex grinder and sieved with a stainless steel sieve. The particles ranging in size between 500 and 1,000 µm were collected as fractions and stored at room temperature (25 ± 5°C) until use.

### 2.3 Sample pretreatment by lyophilization

Greenhouse cultivation of CN1 hybrid and cv. Spunta was carried out in a Tunisian agricultural development company MABROUKA during the period of January–May 2015 (Kammoun et al., 2018). The harvested minitubers were used for field cultures conducted in late season during the period of September–December 2015 in Sfax (Tunisia) using a conventional agronomic method (http://www.ctpta.tn). A number of 100 disease‐free minitubers per field plot were planted. Tubers from the second generation were separately harvested from each plot at their maturity stages, and from a tuber mix, 1 kg of tubers per plot was randomly selected and weighed. The tubers were gently washed, dried with a towel, and peeled. The peel samples were put into plastic bags, rapidly frozen with liquid nitrogen to prevent enzymatic oxidation, and transferred to the freezer (−18°C). Peel sample weight was measured without allowing peels to thaw. The peel samples were then freeze‐dried using a lyophilizer (Telstar LyoQuest); their dry weight was measured, and each sample was separately homogenized for the subsequent phenolic acid and anthocyanin analyses.

### 2.4 Solvent extractions in oven‐dried peels for quantitative analyses of phenolic compounds

Extractions were made for sieved fractions (10 g) either with methanol or water (200 ml) using an overnight treatment at room temperature (20–22°C) with magnetic stirring. The methanol and water extracts were both filtered through a filter paper, and the residues were re‐extracted under the same conditions. The filtrated methanol extracts were combined and then evaporated until dryness in a rotary evaporator at 40°C. The filtrated water extracts were dried through lyophilization (Samarin et al., 2012). The resulting methanol extracts and water extracts were finally weighed and dissolved in distilled water for quantification of phenolic compounds (phenolic acids and anthocyanins) using UHPLC and HPLC.

### 2.5 Determination of phenolic acids

Phenolic acids were analyzed according to the methods described by Mattila et al. (2005), with some modifications. The lyophilized peel samples (500 mg) were extracted with 4 ml of extraction solution (850 ml of methanol + 135 ml of milliQ water + 15 ml of acetic acid + 1.7 g of butylated hydroxy anisole (BHA)) and homogenized with Ultra‐Turrax for the analysis of phenolic acids. Samples were then put into the sonicator for 15 min. After centrifugation, the supernatants were collected into a 10‐ml flask. The extraction procedure was repeated three times. The supernatants of every extraction step were combined into a volume flask and filled up with extraction solvent.

The peel extracts were refiltered (0.2 µm) prior to ultra‐high performance liquid chromatography (UHPLC) analysis. The analytical UHPLC system consists of an Agilent 1290 Infinity Series Ultra‐High Performance Liquid Chromatograph (Agilent Technologies) equipped with a diode array detector. The separation of phenolic acids was performed using a Zorbax Eclipse Plus C18 (2.1 × 50 mm, 1.8 µm) column (Agilent Technologies Inc.) with a C18 guard column. The temperature of the column oven was set at 35°C. A gradient elution was employed with a mobile phase consisting of 50 mM H_3_PO_4_ at pH 2.5 (solvent A) and acetonitrile (solvent B) as follows: isocratic elution 95% A, 0–1.2 min; linear gradient from 95% A to 85% A, 1.2–4.25 min; linear gradient from 85% A to 80% A, 4.25–10 min; linear gradient from 80% A to 50% A, 10–15 min; isocratic elution 50% A, 15–16.2 min; linear gradient from 50% A to 95% A, 16.2–17 min; post time 2 min before the next injection. The injected volume was 2 µl, and the flow rate of the mobile phase was 0.4 ml/min. The UV spectra of the peaks were recorded between 190 and 400 nm, and caffeic and chlorogenic acids were quantified at 329 nm. All quantifications (also for methanol and water extracts, see 2.4.) were based on the peak areas, and the samples were analyzed in triplicate. Chlorogenic acid (5‐CQA) was used as an external standard for all caffeoylquinic acids.

### 2.6 Determination of anthocyanins

Anthocyanins from the peel samples (0.5 g) of the CN1 hybrid were extracted with a 10‐ml extraction solvent (650 ml of methanol + 310 ml of milliQ‐H_2_O + 40 ml of acetic acid) and homogenized with Ultra‐Turrax. The samples were then put into the sonicator for 10 min. After centrifugation, the supernatants were collected into an evaporation pot. The extraction procedure was repeated three times. The extracts were concentrated by evaporation in vacuum (rotavapor) at 40°C and adjusted in the final volume of 2 ml for further analyses.

Anthocyanins were analyzed by Agilent 1100 HPLC device equipped with a diode array detector (DAD) using the method described by Hellström et al. (2013). The extracts were diluted with 5% formic acid (aq) and then filtered through a 0.45 µm membrane filter into HPLC vials. A 150 mm × 4.6 mm i.d., 5 µm, Gemini C18 column with a C‐18 guard column was used for the separation of anthocyanins using a mobile phase that consisted of 5% formic acid and acetonitrile. The temperature of the column oven was set at 35°C, and the flow rate was 1 ml/min. Elution was started with 5% acetonitrile, isocratically 5 min followed by a linear gradient to 13% in 5 min, then a linear gradient to 18% in 10 min, a linear gradient to 80% in 2 min, isocratically 3 min and back to the starting point in 2 min. Post time was 3 min before the next elution. The injected volume of peel extracts was 10 µl. All anthocyanins were quantified (also for methanol and water extracts, see 2.4.) at a detection wavelength of 518 nm using the external standard of cyanidin 3‐O‐glucoside. Three replicates were analyzed from each sample.

### 2.7 Characterization of phenolic acids by LC‐MS

An Acquity UPLC–Xevo G2 QTOF mass spectrometer (Waters, Milford) operated by Waters MassLynx 4.1 software was used for the identification of phenolic acids using the analytical conditions as follows: The compounds were separated on Waters Acquity BEH C18 (1.7 µm, 2.1 mm × 150 mm) column using a gradient of 0.1% formic acid in H_2_O (A) and of 0.1% formic acid in acetonitrile (B). The gradient program was carried out as follows: 2%–60% of B in 24 min, 60%–100% of B in 24–31 min, held at 100% of B for 2 min, 100%–2% in 1 min, and held at 2% of B for 4 min. The flow rate was 0.55 ml/min, temperature of the column oven was 45°C, and the injection volume was 2 µl. An electrospray interface (ESI) was used with capillary voltage of −1 kV in negative mode. The sampling cone was set to 35 V and the extraction cone to 4 V. The cone and desolvation nitrogen gas flows were 15 and 990 L/hr, respectively. The desolvation temperature was 550°C. Source temperature was 150°C. Argon was used as the collision gas. MS analyses were performed by data independent acquisition (*MSE*) centroid data mode in a full scan m/z 50–1200 with 0.2 s scan time. In the *MSE* function, the precursor ions from the low‐collision energy MS mode were fragmented using high collision energy that ramped up from 15 to 40 eV.

### 2.8 Characterization of anthocyanins by LC‐MS

Anthocyanins were characterized using an Acquity Ultra Performance LC interfaced to a Waters Quattro Premier quadruple mass spectrometer (Waters) as described by Gutiérrez‐Quequezana et al. (2020). The separation of the analytes was carried on a Kinetex C18 column (100 × 4.60 mm, 2.6 µm) with a guard column (AJO‐8946, Phenomenex). The flow rate of the mobile phase was 0.5 ml/min, and the injection volume was 10 µl. The mobile phase A was H_2_O/acetonitrile/formic acid (87/3/10) (v/v/v), and mobile phase B contained H_2_O/acetonitrile/ formic acid (40/50/10) (v/v/v). The elution gradient was as follows: 0–20 min 6%–15% B, 25–35 min 20% B, 45–48 min 35% B, 51–53 min 80% B, and 55–65 min 6% B. The parameters of the mass spectrometer were as follows: capillary voltage 3.25 kV, cone voltage 40 V, extractor voltage 3.00 V, source temperature 150°C, desolvation temperature 400°C, cone gas flow 46 L/Hr, and desolvation gas flow 799 L/Hr. The MS analysis (full scan) was performed at a range of m/z 250–1000.

### 2.9 Statistical analyses

The data determined in triplicates were expressed as means ± standard deviation. These data were analyzed statistically by one way ANOVA test using the statistical software GraphPad Prism version 5. Comparisons with *p* values < .05 were considered significantly different.

## 3 RESULTS

### 3.1 Quantities of phenolics in water and methanol extracts

To determine the total quantities of phenolic acids in the peels of CN1 somatic hybrid and cv. Spunta, the comparative extractions were made using water and methanol as solvents. Methanol was clearly more effective than water in the extraction of phenolics, while water extracted much higher amounts of total dry matter than methanol. The extracts of the peels of CN1 hybrid contained higher quantities of phenolic acids than the corresponding extracts of cv. Spunta regardless of whether the extraction solvent was water or methanol. According to the methanol extraction, the peels of CN1 hybrid contained more than threefold of phenolic acids than the peels of cv. Spunta (Table 1.). Methanol was also more effective than water in the extraction of anthocyanins (Table 1.). Anthocyanins were only detected in the extracts of CN1 hybrid since Spunta is a light‐yellow‐skinned cultivar.

**TABLE 1:** The results of the extract yields, content of total phenolic acids (PA) and anthocyanins in water and methanol extracts from oven‐dried peel samples of potato cv. Spunta and CN1 somatic hybrid

	Water extracts		Methanol extracts	
	Spunta	CN1	Spunta	CN1
Recovery of dry matter (%)	23.78^b^ ± 1.19	26.54^a^ ± 1.32	6.00^c^ ± 0.30	7.37^c^ ± 0.37
PA (Chlorogenic acid equivalents, mg/100 g DW)	7.46^c^ ± 0.49	12.30^c^ ± 0.40	27.50^b^ ± 0.70	87.90^a^ ± 6.90
anthocyanins (Cyanidin 3‐glucoside equivalents, mg/100 g DW)	ND	1.15^b^ ± 0.17	ND	3.98^a^ ± 0.26

Note

Abbreviation: ND, not detected.

Each value is presented as the mean ± standard deviation (*n* = 3). Values with the same superscripts within the same line indicate the absence of significant difference (*p* < .05).

### 3.2 Phenolic acids in lyophilized potato peels

**FIGURE 1:**
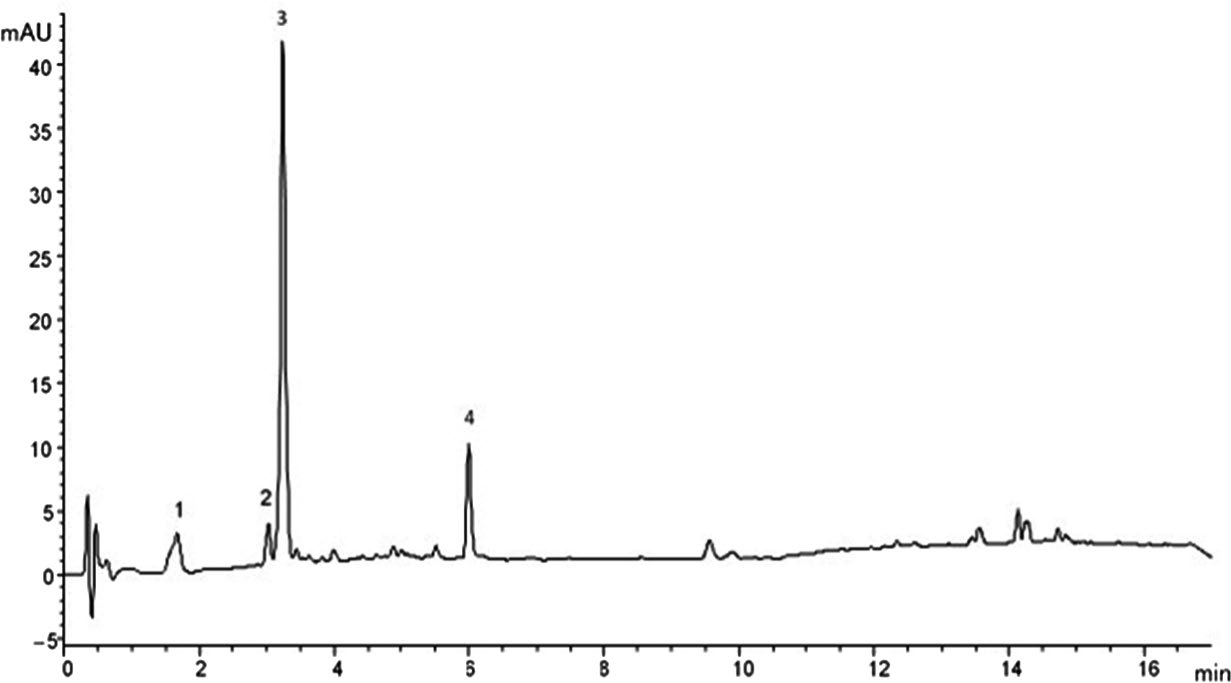
HPLC‐DAD (329 nm) of phenolic acid extract of CN1. Numbers are referring to caffeic acid and its derivatives. 1 = 3‐caffeoylquinic acid, 2 = caffeic acid, 3 = 5‐caffeoylquinic acid, and 4 = 4‐caffeoylquinic acid

Caffeic acid (CA) and three caffeoylquinic acids (CQAs) were identified in peels of both cv. Spunta and CN1 hybrid (Tables 2 and 3). Compound 2 (Figure 1) had the same retention time, UV spectrum and mass (m/z 179) as caffeic acid according to LC‐MS analysis. Compounds 1, 3, and 4 (Figure 1) had similar UV spectra to caffeic acid with a molecular ion at m/z 353 indicating the structure of caffeoylquinic acid. Compounds 1 and 3 had a major fragment at m/z 191 (loss of caffeic acid) followed by m/z 179 (loss of quinic acid) while peak 4 had a major fragment at m/z 173 (loss of caffeic acid and water) followed by m/z 179 and m/z 191. Fragmentation patterns were typical for 3‐CQA, 5‐CQA, and 4‐CQA (Clifford et al., 2003). Ergo, MS data and retention order in the reversed phase column suggested that the compounds 1, 2, 3, and 4 were 3‐CQA, CA, 5‐CQA, and 4‐CQA, respectively. In the CN1 hybrid peels, slightly higher concentrations of phenolic acids (100 mg/100 g DW) were determined than in the corresponding samples of cv. Spunta (83 mg/100 g DW), but the difference was not statistically significant (Table 2.). The predominant isomer of the chlorogenic acids in the peels was 5‐CQA (in CN1 73.7 mg/100 g DW and in Spunta 57.6 mg/100 g DW) (Table 2.). In the cv. Spunta peels, the proportion of different chlorogenic acid isomers (5‐CQA:4‐CQA:3‐CQA:CA) was 70:13:10:7, and the corresponding proportion was 74:14:6:6 in the CN1 peels (Table 2.).

**TABLE 2:** The results of the quantities (mg/100 g DW) of phenolic acids (isomeric forms of caffeoylquinic acids and caffeic acid) and anthocyanins analyzed from lyophilized peel samples of potato cv. Spunta and CN1 somatic hybrid

	3‐CQA	4‐CQA	5‐CQA	CA	Total acids	Anthocyanins
Spunta	8.34 ± 0.34	10.7 ± 0.5	57.6 ± 0.10	6.22 ± 0.10	82.8 ± 0.1	ND
CN1	5.74 ± 2.25	14.7 ± 9.8	73.7 ± 43.9	6.10 ± 2.20	100.2 ± 30.0	5.07 ± 0.25

Note

3‐CQA = 3‐O‐caffeoylquinic acid, 4‐CQA = 4‐O‐caffeoylquinic acid, 5‐CQA = 5‐O‐ caffeoylquinic acid, CA = caffeic acid, ND, not detected.

**TABLE 3:** MS data of phenolic acids and anthocyanins analyzed in the peel samples of potato cultivar Spunta and CN1 somatic hybrid, the major fragments emphasized

Phenolic acids			
Compound number	(M−1)^−^	Fragments	Suggested Compound
1 (Figure 1)	353	179, **191**	3‐Caffeoylquinic acid
2 (Figure 1)	179	‐	Caffeic acid
3 (Figure 1)	353	179, **191**	5‐Caffeoylquinic acid
4 (Figure 1)	353	**173**, 179, 191	4‐Caffeoylquinic acid

Anthocyanins			
Compound number	M+	Fragments	Suggested Compound
1 (Figure 2)	741	271, 433, **579**	pelargonidin−3‐rutinoside−5‐glucoside
2 (Figure 2)	771	301, **609**	peonidin−3‐rutinoside−5‐glucoside
3 (Figure 2)	887	271, 433, **725**	coumaroyl ester of pelargonidin−3‐rutinoside−5‐glucoside **(pelanin)**
4 (Figure 2)	917	301, 464. **755**	coumaroyl ester of peonidin−3‐rutinoside−5‐glucoside
5 (Figure 2)	917	271, 433, **755**	feruloyl ester of pelargonidin−3‐rutinoside−5‐glucoside
6 (Figure 2)	947	301, 464, **785**	feruloyl ester of peonidin−3‐rutinoside−5‐glucoside

### 3.3 Anthocyanins in peels of CN1hybrid

**FIGURE 2:**
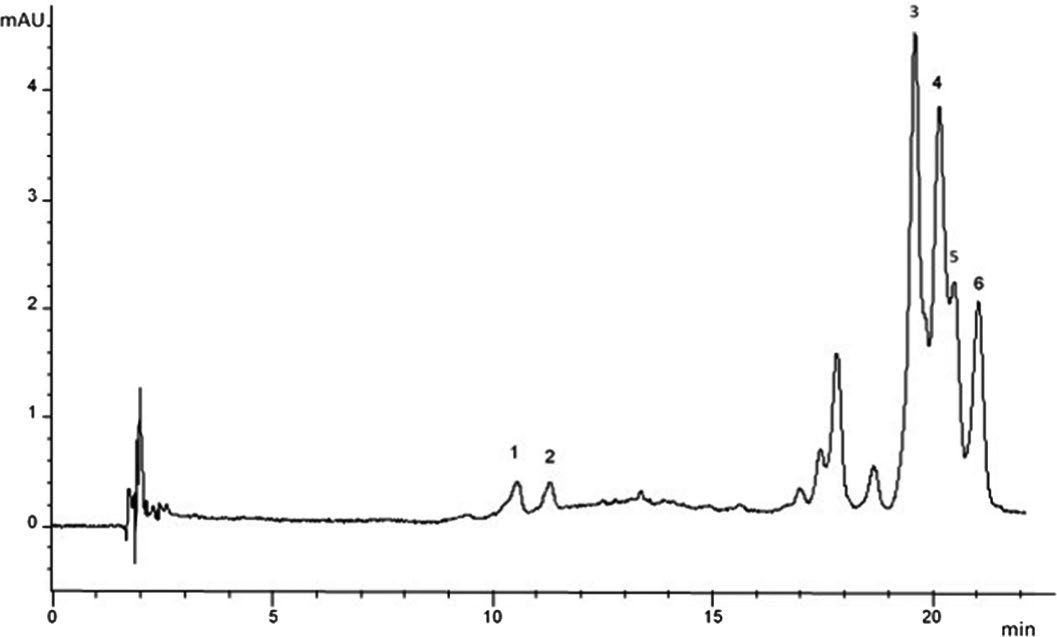
HPLC‐DAD (520 nm) of anthocyanin extract of CN1. Numbers are referring to anthocyanins as follows; 1 = pelargonidin‐3‐rutinoside‐5‐glucoside, 2 = peonidin‐3‐rutinoside‐5‐glucoside, 3 = coumaroyl ester of pelargonidin‐3‐rutinoside‐5‐glucoside, 4 = coumaroyl ester of peonidin‐3‐rutinoside‐5‐glucoside, 5 = feruloyl ester of pelargonidin‐3‐rutinoside‐5‐glucoside, and 6 = feruloyl ester of peonidin‐3‐rutinoside‐5‐glucoside

Lyophilized peels of CN1 hybrid contained 5.07 ± 0.25 mg/100 g DW (as cyanidin 3‐glucoside) of anthocyanins (Table 2.), and six different anthocyanin forms were identified in the same sample (Figure 2, Table 3.). Compound 1 (m/z 741 with fragments at m/z 579; loss of glucose, m/z 433; loss of rutinose, and m/z 271; loss of glucose and rutinose) and compound 2 (m/z 771 with fragments at m/z 609; loss of glucose and m/z 301; loss of glucose and rutinose) were identified as pelargonidin‐3‐rutinoside‐5‐glucoside (Pel‐3‐Rut‐5‐Glc) and peonidin‐3‐rutinoside‐5‐glucoside (Peo‐3‐Rut‐5‐Glc), respectively. Compound 3 (m/z 887 with fragments at m/z 725; loss of glucose, m/z 433; loss of rutinose and coumaric acid, and m/z 271; loss of rutinose, glucose and coumaric acid) and compound 4 (m/z 917 with fragments at m/z 755; loss of glucose, m/z 463; loss of rutinose and coumaric acid, and m/z 301; loss of rutinose, glucose and coumaric acid) were identified as coumaroyl esters of Pel‐3‐Rut‐5‐Glc and Peo‐3‐Rut‐5‐Glc, respectively. Compound 5 (m/z 917 with fragments at m/z 755; loss of glucose, m/z 433; loss of rutinose and ferulic acid, and m/z 271; loss of rutinose, glucose and ferulic acid) and compound 6 (m/z 947 with fragments at m/z 785; loss of glucose, m/z 463; loss of rutinose and glucose, and m/z 301; loss of rutinose, glucose and ferulic acid) were identified as feruloyl esters of Pel‐3‐Rut‐5‐Glc and Peo‐3‐Rut‐5‐Glc, respectively. The UVD at 520 nm (Figure 2.) shows that also some other anthocyanin forms were present, but they could not be identified by LC‐MS due to the coelution of the interfering compounds.

## 4 DISCUSSION

Since the health‐promoting micronutrients are mainly accumulated between the cortex and skin tissues of tubers, and increased quantities of potatoes are peeled for processing industry, the beneficial compounds with an added value are generally disposed of. Processed and fast foods are now increasingly used for nutrition, but yet the generated industrial by‐products are not widely valorized and consumed (Pandey et al., 2009; Schieber & Aranda Saldaña, 2009). Our study focused on characterizing the composition of phenolic compounds in color‐pigmented peels of an intraspecific somatic hybrid of potato. Such potato breeding material produced by protoplast fusion technology has not been previously characterized in terms of the phenolic compound profiles.

There is no universal extraction procedure, which is suitable throughout all the plant phenolics and would be applicable also for nutritional needs (Dai & Mumper, 2010). Standardization is necessary for accurate and reproducible determination of the phenolic compounds from the selected matrices, but the appropriate extracting solvent together with a suitable pretreatment method are not necessarily unambiguous (Albishi et al., 2013; Burgos et al., 2013; Haminiuk et al., 2014). The extraction efficiency of phytochemicals is dependent on the polarity of the extraction solvent, solubility of the compounds, and presence of the interfering substances (Eloff, 1998). The used solvent is one of the most relevant factors together with the extraction time, temperature, sample to solvent ratio, and chemical nature of the sample, which are all influencive on the recoveries and isolation efficiencies (Dai & Mumper, 2010). However, in the practical extraction from industrial by‐products for nutritional applications needs, the solvent must also be amenable to regulatory approval (Wrolstad et al., 2001). Some polyphenolics are water soluble, but solubility is usually better in organic solvents less polar than water (Haminiuk et al., 2014; Shi et al., 2005). In our study, water as an extraction solvent provided higher extract yields of dry matter from potato peels, but methanol extracts contained about fourfold higher quantities of phenolic compounds. Water, however, as a solvent ensures the safety of the extract as a dietary supplement (Shi et al., 2005). Samarin et al. (2012) also concluded that the greatest amount of extract was obtained with water, but methanol extraction provided greatest amount of phenolics. Aqueous extractions are cost‐effective, and they do not leave any harmful residue behind (Shi et al., 2005), but water as a solvent can provide less pure yields (Wrolstad et al., 2001). Therefore, additional steps may still be required to remove the unwanted phenolics and nonphenolic compounds from aqueous extracts (Garcia‐Salas et al., 2010). Ethanol is also considered as a good solvent in terms of the safety of an extract, and improved recovery rates of polyphenols have been obtained using the mixture of water and ethanol (1:1) (Shi et al., 2005).

As described earlier, the pretreatment technique of the plant matrices used for the determination of polyphenols is critical, because it can have effects on the compounds constituent profiles. Polyphenols are widely seen as very unstable and highly susceptible to degradation caused by high temperatures, light, oxygen, and pH (Castañeda‐Ovando et al., 2009). Therefore, in the present study, lyophilized peel samples were applied for profiling phenolic acids and anthocyanins, but this technology cannot be applied for extensive industrial sample amounts, like for waste streams. Abascal et al. (2005) and Gutiérrez‐Quequezana et al. (2018) concluded that freeze‐drying of potato samples was the best pretreatment method for the extraction and retention of anthocyanins.

The amounts of phenolics are known to vary significantly among the different potato varieties (Zhu et al., 2010), and tubers with a pigmented flesh can contain several times higher concentrations of phenolic acids compared to the tubers with white/yellow flesh (Furrer et al., 2017; Lewis et al., 1998). Within potato peels, the differences in total phenolic acid accumulations between red‐skinned (with white flesh) and white/yellow‐skinned potatoes is not that clear (Furrer et al., 2017; Lewis et al., 1998). Deuβer et al. (2012) reported that the peels of red‐skinned cultivars having a white flesh (Cleopatra, Lady Rosetta, Luminella) did not have higher polyphenol contents (phenolic acids, flavanols, flavonols) than the yellow‐skinned cultivars, but the difference with cv. Vitelotte having a purple skin and purple flesh was clear. Similarly, in our study, the phenolic acid levels in the peels of the somatic hybrid CN1 with a pink pigmentation did not noticeably differ from those in yellow peels of cv. Spunta.

Concerning the individual phenolic acids of CN1 hybrid and cv. Spunta, three caffeoylquinic acids (CQAs): 3‐CQA (neochlorogenic acid), 4‐CQA (cryptochlorogenic acid) and 5‐CQA (chlorogenic acid), and free caffeic acid (CA) were identified. The 3‐CQA and CA were, however, considered as minor compounds in both potato genotypes. The total quantity of phenolic acids in peels of the CN1 somatic hybrid was 100 mg/100 g DW, and peels of cv. Spunta had almost a similar amount (83 mg/100 g DW). Previously, Deuβer et al. (2012) had quantified the total amount of 196 mg/100 g DW of phenolic acids in cv. Spunta's peels. The predominant isomer of the chlorogenic acids in the peels was 5‐CQA (70%–74%), which is in line with other reports, describing the 5‐CQA ratio of 50%–85% in whole tubers (Furrer et al., 2017; Griffiths & Bain, 1997; Navarre et al., 2011), or in potato peels (Mäder et al., 2009). The 4‐CQA is generally more abundant than 3‐CQA in unpeeled tubers (Navarre et al., 2011), and the 4‐CQA levels in peels may gain almost the ratio of 5‐CQA (Deuβer et al., 2012). The environmental growth conditions (Andre et al., 2009), physiological stage of tubers at harvest (Leja, 1989), and tuber storage conditions (Griffiths et al., 1995) have a significant effect on the total phenolic contents in potatoes. In our study, the proportion of different chlorogenic acid isomers (5‐CQA:4‐CQA:3‐CQA:CA) was 70:13:10:7 in the peels of cv. Spunta cultivated in Tunisia, but the peels of the corresponding cultivar cultivated in Luxembourg had the proportion of 47:29:19:5 (Deuβer et al., 2012). The difference in profiles of individual chlorogenic acid isomers might be due to differences in growth conditions, but also to postharvest handling. For example, exposure to light and higher pH values can cause isomerization by increasing the concentrations of 3‐CQA and 4‐CQA, but decrease the level of 5‐CQA (Griffiths & Bain, 1997). Similarly, extracts from potato peels exposed to light and stored at 25°C have shown degradation of chlorogenic acids to caffeic acid (Rodriguez de Sotillo et al., 1994).

The anthocyanin content in the CN1 hybrid peels was 5.07 ± 0.25 mg/100 g DW. Nemś et al. (2015) and Gutiérrez‐Quequezana et al. (2018) quantified the total anthocyanin concentrations to range between 39 and 318 mg/100 g DW in potato varieties with blue‐purple‐colored flesh and skin. Generally, the total anthocyanin levels in unpeeled whole tubers with pigmented flesh are less than 40 mg/100 g FW (Brown, 2005). The concentrations of anthocyanins are higher in colored skins (<700 mg /100 g FW) than in colored flesh (<200 mg/ 100 g FW) (Lewis et al., 1998), but these high values were detected only in specific potato genotypes. The relatively low anthocyanin concentration in CN1 hybrid peels is due to the considerably low color pigmentation intensity with white flushes in the skin. The color pigmentation can, however, be induced through changing environmental factors, such as higher light intensity, lower growth temperature, drier soil, higher nitrogen, and by harvesting tubers at later maturity stages (Lewis et al., 1999; Reyes et al., 2004). The storage conditions can also induce the concentration of particular anthocyanin derivate, but in parallel decrease the levels of the other compounds (Rodriguez‐Saona et al., 1998). In CN1 hybrid, it was demonstrated that growth season had an influence on the intensity of red pigmentation (Kammoun et al., 2018), which would be a benefit for industrial production of these compounds.

Mori et al. (2010) reported that the red tuber skin in cv. Desirée had the same anthocyanin composition as found in the present study, but CN1 has higher peonidin derivate ratios compared to pelanin and other pelargonidin derivates. In certain red‐fleshed cultivars, pelargonidin derivatives can be the only anthocyanins detected, mainly in their acylated forms (Nemś et al., 2015), but often traces or even equal amounts of peonidin derivates can be present (Brown, 2005; Lachman et al., 2012; Rodriguez‐Saona et al., 1998). The CN1 anthocyanin profile resembles the corresponding composition of red‐skinned Japanese Type C potatoes (Mori et al., 2010). Cardinal, the CN1 hybrid parent, is a potato variety with a red skin and a pink sprout color (https://www.europotato.org), and crossing red and white‐skinned parents can produce pink‐skinned progeny (Van Eck et al., 1994). According to our findings, the peel waste derived from CN1 hybrid may have an added commercial value based on its specific anthocyanin profile. In processing technology, the stability of anthocyanins varies between potato cultivars (Lachman et al., 2012), and the most stable anthocyanins in thermal processes are known to be pelargonidin derivatives and their acyl conjugates (Nemś et al., 2015; Qian et al., 2017), which are also common in the peels of CN1 hybrid. Pigments of red potatoes can be used as natural food colorants since they are also soluble in water (Rodriguez‐Saona et al., 1998), and the colors in red potatoes are more stable than those in purple potatoes (Nemś et al., 2015). The advantage of the red‐colored potatoes is not only the stability of the red pigments, but also the attractive red hue with a high intensity (Wrolstad et al., 2001) (U.S. patent). The major anthocyanins in grapes and certain berries are nonacylated, and therefore, the stability of the pigments derived from berries after extraction is a limiting factor as a food colorant (Rein & Heinonen, 2004; Rodriguez‐Saona et al., 1998).

According to FAO, about 1.3 Mt (one third) of the produced food is wasted every year throughout the whole value chain from farmers to consumers in the world (Fritsch et al., 2017). In potato processing, the industry generates between 70,000 and 140,000 tons of peels worldwide annually (Wu, 2016). Analyzing the nutritional content of the by‐products is highly relevant, if the by‐products are used for food or feed. Since potato is a low‐cost crop in terms of production, the pigmented potatoes (like CN1) may serve as a potential source of natural anthocyanins for the development of health‐promoting food ingredients in the future. Through potato breeding, it is also possible to develop cultivars with higher pigment levels to make them more suitable for production of value‐added compounds.

## CONFLICT OF INTEREST

The authors declare that they have no known competing financial interests or personal relationships that could have appeared to influence the work reported in this paper.

## Data Availability

The data that support the findings of this study are openly available in [repository name] at http://doi.org/[doi].

## References

[fsn32100-bib-0001] Abascal, K. , Ganora, L. , & Yarnell, E. (2005). The effect of freeze‐drying and its implications for botanical medicine: A review. Phytotherapy Research, 19, 655–660. 10.1002/ptr.1651. 16177965

[fsn32100-bib-0002] Albishi, T. , John, J. A. , Al‐Khalifa, A. S. , & Shahidi, F. (2013). Phenolic content and antioxidant activities of selected potato varieties and their processing by‐products. Journal of Functional Foods, 5, 590–600. 10.1016/j.jff.2012.11.019

[fsn32100-bib-0003] Amiot, M. J. , Riva, C. , & Vinet, A. (2016). Effects of dietary polyphenols on metabolic syndrome features in humans: A systematic review. Obesity Reviews, 17, 573–586. 10.1111/obr.12409 27079631

[fsn32100-bib-0004] Andre, C. M. , Oufir, M. , Guignard, C. , Hoffmann, L. , Hausman, J.‐F. , Evers, D. , & Larondelle, Y. (2007). Antioxidant profiling of native Andean potato tubers (*Solanum tuberosum* L.) reveals cultivars with high levels of β‐carotene, α‐tocopherol, chlorogenic acid, and petanin. Journal of Agricultural and Food Chemistry, 55, 10839–10849. 10.1021/jf0726583 18044831

[fsn32100-bib-0005] Andre, C. M. , Oufir, M. , Hoffmann, L. , Hausman, J.‐F. , Rogez, H. , Larondelle, Y. , & Evers, D. (2009). Influence of environment and genotype on polyphenol compounds and in vitro antioxidant capacity of native Andean potatoes (*Solanum tuberosum* L.). Journal of Food Composition and Analysis, 22, 517–524. 10.1016/j.jfca.2008.11.010

[fsn32100-bib-0006] Birch, P. R. J. , Bryan, G. , Fenton, B. , Gilroy, E. M. , Hein, I. , Jones, J. T. , Prashar, A. , Taylor, M. A. , Torrance, L. , & Toth, I. K. (2012). Crops that feed the world 8. Potato: Are the trends of increased global production sustainable? Food Security, 4, 477–508. 10.1007/s12571-012-0220-1

[fsn32100-bib-0007] Brat, P. , Georgé, S. , Bellamy, A. , Du Chaffaut, L. , Scalbert, A. , Mennen, L. , Arnault, N. , & Amiot, M. J. (2006). Daily polyphenol intake in France from fruit and vegetables. Journal of Nutrition, 136, 2368–2373. 10.1093/jn/136.9.2368 16920856

[fsn32100-bib-0008] Brown, C. R. (2005). Antioxidants in potato. American Journal of Potato Research, 82, 163–172. 10.1007/BF02853654

[fsn32100-bib-0009] Burgos, G. , Amoros, W. , Muñoa, L. , Sosa, P. , Cayhualla, E. , Sanchez, C. , Díaz, C. , & Bonierbale, M. (2013). Total phenolic, total anthocyanin and phenolic acid concentrations and antioxidant activity of purple‐fleshed potatoes as affected by boiling. Journal of Food Composition and Analysis, 30, 6–12. 10.1016/j.jfca.2012.12.001

[fsn32100-bib-0010] Castañeda‐Ovando, A. , Pacheco‐Hernández, M. L. , Páez‐Hernández, M. E. , Rodríguez, J. A. , & Galán‐Vidal, C. A. (2009). Chemical studies of anthocyanins: A review. Food Chemistry, 113, 859–871. 10.1016/j.foodchem.2008.09.001

[fsn32100-bib-0011] Chen, A. Y. , & Chen, Y. C. (2013). A review of the dietary flavonoid, kaempferol on human health and cancer chemoprevention. Food Chemistry, 138, 2099–2107. 10.1016/j.foodchem.2012.11.139 23497863 PMC3601579

[fsn32100-bib-0012] Cheynier, V. (2005). Polyphenols in foods are more complex than often thought. The American Journal of Clinical Nutrition, 81, 223S–S229. 10.1093/ajcn/81.1.223S 15640485

[fsn32100-bib-0013] Chun, O. K. , Kim, D. O. , Smith, N. , Schroeder, D. , Han, J. T. , & Lee, C. Y. (2005). Daily consumption of phenolics and total antioxidant capacity from fruit and vegetables in the American diet. Journal of the Science of Food and Agriculture, 85, 1715–1724. 10.1002/jsfa.2176

[fsn32100-bib-0014] Clifford, M. N. (2000). Chlorogenic acids and other cinnamates – nature, occurrence, dietary burden, absorption and metabolism. Journal of the Science of Food and Agriculture, 80, 1033–1043. 10.1002/(SICI)1097-0010(20000515)80:7<1033:AID-JSFA595>3.0.CO;2-T

[fsn32100-bib-0015] Clifford, M. N. , Johnston, K. L. , Knight, S. , & Kuhnert, N. (2003). Hierarchical scheme for LC‐MS*^n^* identification of chlorogenic acids. Journal of Agricultural and Food Chemistry, 51, 2900–2911. 10.1021/jf026187q 12720369

[fsn32100-bib-0016] Dai, J. , & Mumper, R. J. (2010). Plant phenolics: Extraction, analysis and their antioxidant and anticancer properties. Molecules, 15, 7313–7352. 10.3390/molecules15107313 20966876 PMC6259146

[fsn32100-bib-0017] del Mar Verde Méndez, C. , Rodríguez Delgado, M. Á. , Rodríguez Rodríguez, E. M. , & Díaz Romero, C. (2004). Content of free phenolic compounds in cultivars of potatoes harvested in Tenerife (Canary Islands). Journal of Agricultural and Food Chemistry, 2004, 1323–1327. 10.1021/jf0345595 14995140

[fsn32100-bib-0018] Deuβer, H. , Guignard, C. , Hoffmann, L. , & Evers, D. (2012). Polyphenol and glycoalkaloid contents in potato cultivars grown in Luxembourg. Food Chemistry, 135, 2814–2824. 10.1016/j.foodchem.2012.07.028 22980877

[fsn32100-bib-0019] Eloff, J. N. (1998). Which extractant should be used for the screening and isolation of antimicrobial components from plants? Journal of Ethnopharmacology, 60, 1–8. 10.1016/s0378-8741(97)00123-2 9533426

[fsn32100-bib-0020] Ezekiel, R. , Singh, N. , Sharma, S. , & Kaur, A. (2013). Beneficial phytochemicals in potato — a review. Food Research International, 50, 487–496. 10.1016/j.foodres.2011.04.025

[fsn32100-bib-0021] FAOSTAT (2020). Food and Agriculture Organization of the United Nations. http://www.fao.org/FAOSTAT./en/#data.

[fsn32100-bib-0022] Fossen, T. , & Andersen, Ø. M. (2000). Anthocyanins from tubers and shoots of the purple potato, *Solanum tuberosum* . The Journal of Horticultural Science and Biotechnology, 75, 360–363. 10.1080/14620316.2000.11511251

[fsn32100-bib-0023] Fossen, T. , Øvstedal, D. O. , Slimestad, R. , & Andersen, Ø. M. (2003). Anthocyanins from a Norwegian potato cultivar. Food Chemistry, 81, 433–437. 10.1016/S0308-8146(02)00473-9

[fsn32100-bib-0024] Friedman, M. (1997). Chemistry, biochemistry, and dietary role of potato polyphenols. A review. Journal of Agricultural and Food Chemistry, 45, 1523–1540. 10.1021/jf960900s

[fsn32100-bib-0025] Friedman, M. (2006). Potato glycoalkaloids and metabolites: Roles in the plant and in the diet. Journal of Agricultural and Food Chemistry, 54, 8655–8681. 10.1021/jf061471t 17090106

[fsn32100-bib-0026] Friedman, M. , Roitman, J. N. , & Kozukue, N. (2003). Glycoalkaloid and calystegine contents of eight potato cultivars. Journal of Agricultural and Food Chemistry, 51, 2964–2973. 10.1021/jf021146f 12720378

[fsn32100-bib-0027] Fritsch, C. , Staebler, A. , Happel, A. , Márquez, M. A. C. , Aguiló‐Aguayo, I. , Abadias, M. , Gallur, M. , Cigognini, I. M. , Montanari, A. , López, M. J. , Suárez‐Estrella, F. , Brunton, N. , Luengo, E. , Sisti, L. , Ferri, M. , & Belotti, G. (2017). Processing, valorization and application of bio‐waste derived compounds from potato, tomato, olive and cereals: A review. Sustainability, 9, 1492. 10.3390/su9081492

[fsn32100-bib-0028] Furrer, A. , Cladis, D. P. , Kurilich, A. , Manoharan, R. , & Ferruzzi, M. G. (2017). Changes in phenolic content of commercial potato varieties through industrial processing and fresh preparation. Food Chemistry, 218, 47–55. 10.1016/j.foodchem.2016.08.126 27719937

[fsn32100-bib-0029] Garcia‐Salas, P. , Morales‐Soto, A. , Segura‐Carretero, A. , & Fernández‐Gutierréz, A. (2010). Phenolic‐compound‐extraction systems for fruit and vegetable samples. Molecules, 15, 8813–8826. 10.3390/molecules15128813 21131901 PMC6259353

[fsn32100-bib-0030] Griffiths, D. W. , & Bain, H. (1997). Photo‐induced changes in the concentrations of individual chlorogenic acid isomers in potato (*Solanum tuberosum*) tubers and their complexation with ferric acid. Potato Research, 40, 307–315. 10.1007/BF02358012

[fsn32100-bib-0031] Griffiths, D. W. , Bain, H. , & Dale, M. F. B. (1995). Photo‐induced changes in total chlorogenic acid content of potato (*Solanum tuberosum*) tubers. Journal of the Science of Food and Agriculture, 68, 105–110. 10.1002/jsfa.2740680117

[fsn32100-bib-0032] Gutiérrez‐Quequezana, L. , Vuorinen, A. L. , Kallio, H. , & Yang, B. (2018). Improved analysis of anthcyanins and vitamin C in blue‐purple potato cultivars. Food Chemistry, 242, 217–224. 10.1016/j.foodchem.2017.09.002 29037681

[fsn32100-bib-0033] Gutiérrez‐Quequezana, L. , Vuorinen, A. L. , Kallio, H. , & Yang, B. (2020). Impact of cultivar, growth temperature and developmental stage on phenolic compounds and ascorbic acid in purple and yellow potato tubers. Food Chemistry, 326, 126966. 10.1016/j.foodchem.2020.126966 32416419

[fsn32100-bib-0034] Haminiuk, C. W. I. , Plata‐Oviedo, M. S. V. , de Mattos, G. , Carpes, S. T. , & Branco, I. G. (2014). Extraction and quantification of phenolic acids and flavonols from *Eugenia pyriformis* using different solvents. Journal of Food Science and Technology, 51, 2862–2866. 10.1007/s13197-012-0759-z 25328239 PMC4190214

[fsn32100-bib-0035] Harborne, J. B. (1960). Plant polyphenols. 1. Anthocyanin production in the cultivated potato. Biochemical Journal, 74, 262–269. 10.1042/bj0740262 PMC1204152 14399694

[fsn32100-bib-0036] Hellström, J. , Mattila, P. , & Karjalainen, R. (2013). Stability of anthocyanins in berry juices stored at different temperatures. Journal of Food Composition and Analysis, 31, 12–19. 10.1016/j.jfca.2013.02.010

[fsn32100-bib-0037] Kammoun, M. , Bouallous, O. , Ksouri, M. F. , Gargouri‐Bouzid, R. , & Nouri‐Ellouz, O. (2018). Agro‐physiological and growth response to reduced water supply of somatic hybrid potato plants (*Solanum tuberosum* L.) cultivated under greenhouse conditions. Agricultural Water Management, 203, 9–19. 10.1016/j.agwat.2018.02.032

[fsn32100-bib-0038] Lachman, J. , Hamouz, K. , Orsák, M. , Pivec, V. , Hejtmánková, K. , Pazderů, K. , Dvořák, P. , & Čepl, J. (2012). Impact of selected factors – Cultivar, storage, cooking and baking on the content of anthocyanins in coloured‐flesh potatoes. Food Chemistry, 133, 1107–1116. 10.1016/j.foodchem.2011.07.077

[fsn32100-bib-0039] Lattanzio, V. , Lattanzio, V. M. T. , & Cardinali, A. (2006). Role of phenolics in the resistance mechanisms of plants against fungal pathogens and insects. In F. Imperato (Ed.), Phytochemistry: Advances in research. (pp. 23–67), : Research Signpost.

[fsn32100-bib-0040] Leja, M. (1989). Chlorogenic acid as the main phenolic compound of mature and immature potato tubers stored at low and high temperature. Acta Physiologiae Plantarum, 11, 201–206.

[fsn32100-bib-0041] Lewis, C. E. , Walker, J. R. L. , & Lancaster, J. E. (1999). Changes in anthocyanin, flavonoid, and phenolic acid concentrations during development and storage of coloured potato (*Solanum tuberosum* L) tubers. Journal of the Science of Food and Agriculture, 79, 311–316. 10.1002/(SICI)1097-0010(199902)79:2<311:AID-JSFA199>3.0.CO;2-Q

[fsn32100-bib-0042] Lewis, C. E. , Walker, J. R. L. , Lancaster, J. E. , & Sutton, K. H. (1998). Determination of anthocyanins, flavonoids and phenolic acids in potatoes. I: Coloured cultivars of *Solanum tuberosum* L. Journal of the Science of Food and Agriculture, 77, 45–57. 10.1002/(SICI)1097-0010(199805)77:1<45:AID-JSFA1>3.0.CO;2-S

[fsn32100-bib-0043] Loo, B. M. , Erlund, I. , Koli, R. , Puukkaa, P. , Hellström, J. , Wähäläf, K. , Mattila, P. , & Jula, A. (2016). Consumption of chokeberry (*Aronia mitschurinii*) products modestly lowered blood pressure and reduced low‐grade inflammation in patients with mildly elevated blood pressure. Nutrition Research, 36, 1222–1230. 10.1016/j.nutres.2016.09.005 27865620

[fsn32100-bib-0044] Mäder, J. , Rawel, H. , & Kroh, L. W. (2009). Composition of phenolic compounds and glycoalkaloids α‐solanine and α‐chaconine during commercial potato processing. Journal of Agricultural and Food Chemistry, 57, 6292–6297. 10.1021/jf901066k 19534529

[fsn32100-bib-0045] Malmberg, A. G. , & Theander, O. (1985). Determination of chlorogenic acid in potato tubers. Journal of Agricultural and Food Chemistry, 33, 549–551. 10.1021/jf00063a052

[fsn32100-bib-0046] Manach, C. , Scalbert, A. , Morand, C. , Rémésy, C. , & Jiménez, L. (2004). Polyphenols: Food sources and bioavailability. The American Journal of Clinical Nutrition, 79, 727–747. 10.1093/ajcn/79.5.727 15113710

[fsn32100-bib-0047] Mattila, P. , & Hellström, J. (2007). Phenolic acids in potatoes, vegetables, and some of their products. Journal of Food Composition and Analysis, 20, 152–160. 10.1016/j.jfca.2006.05.007

[fsn32100-bib-0048] Mattila, P. , Pihlava, J.‐M. , & Hellström, J. (2005). Contents of phenolic acids, alkyl‐ and alkenylresorcinols, and avenanthramides in commercial grain products. Journal of Agricultural and Food Chemistry, 53, 8290–8295. 10.1021/jf051437z 16218677

[fsn32100-bib-0049] Medina‐Remón, A. , Tresserra‐Rimbau, A. , Pons, A. , Tur, J. A. , Martorell, M. , Ros, E. , Buil‐Cosiales, P. , Sacanella, E. , Covas, M. I. , Corella, D. , Salas‐Salvadó, J. , Gómez‐Gracia, E. , Ruiz‐Gutiérrez, V. , Ortega‐Calvo, M. , García‐Valdueza, M. , Arós, F. , Saez, G. T. , Serra‐Majem, L. , Pinto, X. , … Lamuela‐Raventos, R. M. (2015). Effects of total dietary polyphenols on plasma nitric oxide and blood pressure in a high cardiovascular risk cohort. The PREDIMED randomized trial. Nutrition, Metabolism, and Cardiovasularc Diseases, 25, 60–67. 10.1016/j.numecd.2014.09.001 25315667

[fsn32100-bib-0050] Mori, M. , Hayashi, K. , Ohara‐takada, A. , Watanuki, H. , Katahira, R. , Ono, H. , & Terahara, N. (2010). Anthocyanins from skins and fleshes of potato varieties. Food Science and Technology Research, 16, 115–122. 10.3136/fstr.16.115

[fsn32100-bib-0051] Murashige, T. , & Skoog, F. (1962). A revised medium for rapid growth and bio assays with tobacco tissue cultures. Physiologia Plantarum, 15, 473–497. 10.1111/j.1399-3054.1962.tb08052.x

[fsn32100-bib-0052] Navarre, D. A. , Pillai, S. S. , Shakya, R. , & Holden, M. J. (2011). HPLC profiling of phenolics in diverse genotypes. Food Chemistry, 127, 34–41. 10.1016/j.foodchem.2010.12.080

[fsn32100-bib-0053] Nemś, A. , Pęksa, A. , Kucharska, A. Z. , Sokół‐Łętowska, A. , Kita, A. , Drożdż, W. , & Hamouz, K. (2015). Anthocyanin and antioxidant activity of snacks with coloured potato. Food Chemistry, 172, 175–182. 10.1016/j.foodchem.2014.09.033 25442540

[fsn32100-bib-0054] Nouri‐Ellouz, O. , Gargouri‐Bouzid, R. , Sihachakr, D. , Triki, M. A. , Ducreux, G. , Drira, N. , & Lakhoua, L. (2006). Production of potato intraspecific somatic hybrids with improved tolerance to PVY and *Pythium aphanidermatum* . Journal of Plant Physiology, 163, 1321–1332. 10.1016/j.jplph.2006.06.009 16904234

[fsn32100-bib-0055] Ochiai, R. , Sugiura, Y. , Otsuka, K. , Katsuragi, Y. , & Hashiguchi, T. (2015). Coffee bean polyphenols ameliorate postprandial endothelial dysfunction in healthy male adults. International Journal of Food Sciences and Nutrition, 66, 350–354. 10.3109/09637486.2015.1007453 25666414

[fsn32100-bib-0056] Pandey, S. K. , Marwaha, R. S. , Kumar, D. , & Singh, S. V. (2009). Indian potato processing story: Industrial limitations, challenges ahead and vision for the future. Potato Journal, 36, 1–13.

[fsn32100-bib-0057] Qian, B. J. , Liu, J. H. , Zhao, S. J. , Cai, J. X. , & Jing, P. (2017). The effects of gallic/ferulic/caffeic acids on colour intensification and anthocyanin stability. Food Chemistry, 228, 526–532. 10.1016/j.foodchem.2017.01.120. 28317759

[fsn32100-bib-0058] Reddivari, L. , Vanamala, J. , Chintharlapalli, S. , Safe, S. H. , & Miller, J. C. Jr (2007). Anthocyanin fraction from potato extracts is cytotoxic to prostate cancer cells through activation of caspase‐dependent and caspase‐independent pathways. Carcinogenesis, 28, 2227–2235. 10.1093/carcin/bgm117 17522067

[fsn32100-bib-0059] Rein, M. , & Heinonen, M. (2004). Stability and enhancement of berry juice color. Journal of Agricultural and Food Chemistry, 52, 3106–3114. 10.1021/jf035507i 15137861

[fsn32100-bib-0060] Reyes, L. F. , Miller, J. C. Jr , & Cisneros‐Zevallos, L. (2004). Environmental conditions influence the content and yield of anthocyanins and total phenolics in purple‐ and red‐flesh potatoes during tuber development. American Journal of Potato Research, 81, 187–193. 10.1007/BF02871748

[fsn32100-bib-0061] Rodriguez de Sotillo, D. , Hadley, M. , & Holm, E. T. (1994). Phenolics in aqueous potato peel extract: Extraction, identification and degradation. Journal of Food Science, 59, 649–651. 10.1111/j.1365-2621.1994.tb05584.x

[fsn32100-bib-0062] Rodriguez‐Saona, L. E. , Giusti, M. M. , & Wrolstad, R. E. (1998). Anthocyanin pigment compositions of red‐fleshes potatoes. Journal of Food Science, 63, 458–465. 10.1111/j.1365-2621.1998.tb15764.x

[fsn32100-bib-0063] Ross, J. A. , & Kasum, C. M. (2002). Dietary flavonoids: Bioavailability, metabolic effects, and safety. Annual Review of Nutrition, 22, 193–194. 10.1146/annurev.nutr.22.111401.144957 12055336

[fsn32100-bib-0064] Samarin, A. M. , Poorazarang, H. , Hematyar, N. , & Elhamirad, A. (2012). Phenolics in potato peels: Extraction and utilization as natural antioxidants. World Applied Sciences Journal, 18, 191–195. 10.5829/idosi.wasj.2012.18.02.1057

[fsn32100-bib-0065] Schieber, A. , & Aranda Saldaña, M. D. (2009). Potato peels: A source of nutritionally and pharmacologically interesting compounds – a review. Food, 3, 23–29.

[fsn32100-bib-0066] Shi, J. , Nawaz, H. , Pohorly, J. , Mittal, G. , Kakuda, Y. , & Jiang, Y. (2005). Extraction of polyphenolics from plant material for functional foods‐engineering and technology. Food Reviews International, 21, 139–166. 10.1081/FRI-200040606

[fsn32100-bib-0067] Van Eck, H. J. , Jacobs, J. M. E. , van den Berg, P. M. M. M. , Stiekema, W. J. , & Jacobsen, E. (1994). The inheritance of anthocyanin pigmentation in potato (*Solanum tuberosum* L.) and mapping of the tuber skin colour loci using RFLPs. Heredity, 73, 410–421. 10.1038/hdy.1994.189

[fsn32100-bib-0068] Wrolstad, R. E. , Giusti, M. M. , Rodriguez‐Saona, L. E. , & Durst, R. W. (2001). Anthocyanins from radishes and red‐fleshed potatoes. In J. M. Ames , & T. Hofmann (Eds.), Chemistry and physiology of selected food colorants. ACS Symposium Series, American Chemical Society.

[fsn32100-bib-0069] Wu, D. (2016). Recycle technology for potato peel waste processing: A review. Procedia Environnemental Sciences, 31, 103–107. 10.1016/j.proenv.2016.02.014

[fsn32100-bib-0070] Yamagata, K. , Tagami, M. , & Yamori, Y. (2015). Dietary polyphenols regulate endothelial function and prevent cardiovascular disease. Nutrition, 31, 28–37. 10.1016/j.nut.2014.04.011. 25466651

[fsn32100-bib-0071] Zhu, F. , Cai, Y.‐Z. , Ke, J. , & Corke, H. (2010). Compositions of phenolic compounds, amino acids and reducing sugars in commercial potato varieties and their effects on acrylamide formation. Journal of the Science of Food and Agriculture, 90, 2254–2262. 10.1002/jsfa.4079 20629114

